# Effect of olive oil phenols on oxidative stress biomarkers: A systematic review and dose–response meta‐analysis of randomized clinical trials

**DOI:** 10.1002/fsn3.3251

**Published:** 2023-03-13

**Authors:** Seyedeh‐Masomeh Derakhshandeh‐Rishehri, Asma Kazemi, Sung Ryul Shim, Mostafa Lotfi, Shabnam Mohabati, Mehran Nouri, Shiva Faghih

**Affiliations:** ^1^ DONALD Study Centre University of Bonn Dortmund Germany; ^2^ Nutrition Research Center Shiraz University of Medical Sciences Shiraz Iran; ^3^ Department of Preventive Medicine Korea University College of Medicine Seoul South Korea; ^4^ Department of Community Nutrition, School of Nutrition and Food Sciences Shiraz University of Medical Sciences Shiraz Iran; ^5^ Student Research Committee Shiraz University of Medical Sciences Shiraz Iran; ^6^ Health Policy Research Center, Institute of Health Shiraz University of Medical Sciences Shiraz Iran

**Keywords:** dose–response, low density lipoprotein cholesterol, malondialdehyde, olive oil, oxidative stress

## Abstract

The phenolic content of olive oil has a role in cardiovascular protection. Some clinical trial studies demonstrated that phenolic compounds of olive oil have antioxidant activity which can protect macronutrients from oxidative damages. The aim of this study was to summarize the results of clinical trials which assessed the effects of high‐ versus low‐phenol olive oil on oxidative stress biomarkers levels. We searched Scopus, PubMed, Web of Science, Google Scholar, ProQuest, and Embase up to July 2021. Eight clinical trials which evaluated the effect of the phenolic content of olive oil on oxidized‐LDL (ox‐LDL), malondialdehyde (MDA), or ferric‐reducing ability of plasma (FRAP) were included the meta analysis. A significant decrease was observed in ox‐LDL level (WMD: −0.29 U/L; 95% CI: −0.51, −0.07) and MDA (WMD: −1.82 μmoL/L; 95% CI: −3.13, −0.50). However, after subgroup analysis for MDA, the result was not significant for not serious limitation (SMD: −0.05, 95% CI: −0.35 to 0.24), but significant for serious limitation (SMD: −3.64, 95% CI: −4.29 to −2.99). Also, no significant change was found in FRAP (WMD: 0.0 mmoL/L; 95% CI: −0.03, 0.04) level. Dose–response analysis indicated a significant linear relationship between the phenolic content of olive oil and ox‐LDL. The present study showed some beneficial effects of high‐phenol compared with low‐phenol olive oil on ox‐LDL and MDA levels. According to the meta‐regression analysis along with the increasing phenolic content of olive oil, a reduction in oxidative stress biomarkers was observed.

## Graphical abstract

The aim of this study was to summarize the results of clinical trials which assessed the effects of high‐ versus low‐phenol olive oil on oxidative stress biomarkers level. We searched Scopus, PubMed, Web of Science, Google Scholar, ProQuest, and Embase up to July 2021. Dose–response analysis indicated a significant linear relationship between the phenolic content of olive oil and Ox‐LDL. The present study showed some beneficial effects of high‐phenol compared with the low‐phenol olive oil on Ox‐LDL and MDA levels.

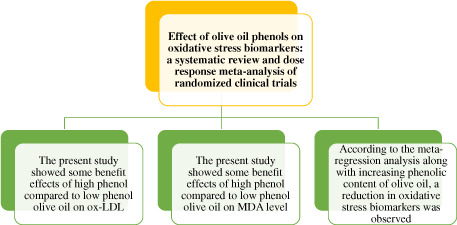

## 1 INTRODUCTION

Mediterranean diet or diets rich in olive oil are associated with lower risk of chronic diseases such as coronary heart disease, hypertension, type II diabetes mellitus, and cancer (Beauchamp et al., 2005; Farràs et al., 2015; Perona et al., 2004). As the main ingredient of the Mediterranean diet, virgin olive oil consists of oleic acid and about 500 mg/L of polyphenols, and extra virgin olive oil (EVOO) has an exclusive polyphenol composition including hydroxytyrosol and oleuropei (Mataix et al., 2006). In various studies, favorable effects of these ingredients have been demonstrated as modulation of pathways related to inflammation, oxidative stress, and cell adhesion (Parkinson & Cicerale, 2016; Peyrol et al., 2017).

Although reactive oxygen species (ROSS) are synthesized via different aerobic pathways, the main source of ROS is mitochondria (Mataix et al., 2006). About 1–5 percent of oxygen which is consumed by mitochondria is not fully converted to water. In turn, these oxygens are more converted to ROSs such as superoxide anions (Mataix et al., 2006). It is proved that monounsaturated fatty acids cause more protection against oxidative stress than polyunsaturated fatty acids (Mataix et al., 2006).

Some clinical trials demonstrated that phenolic compounds of olive oil have antioxidant activity which can protect DNA, lipids, and proteins from ROS damages (Fki et al., 2007). Previous studies have shown the beneficial effects of virgin olive oil on oxidative stress–related diseases, such as fibromiolgia (Rus et al., 2016), cardiovascular diseases (Guasch‐Ferré et al., 2014), rheumatoid arthritis (Berbert et al., 2005), and cancer (Pelucchi et al., 2011).

To investigate the role of phenol‐rich olive oils on oxidative stress biomarkers, various studies have assessed the effect of high‐polyphenol versus low‐polyphenol olive oils. Since, no systematic reviews and meta‐analysis have assessed the effects of the phenolic content of olive oil on oxidative stress biomarkers, the aim of the present systematic review and meta‐analysis was to examine the effects of the phenolic content of olive oil on biomarkers of oxidative stress in adults.

## 2 MATERIALS AND METHODS

The present systematic review and meta‐analysis were registered in PROSPERO, https://www.crd.york.ac.uk/PROSPERO [PROSPERO registration number: CRD42021268421].

### 2.1 Search strategy

To find relevant papers, we searched Scopus, PubMed, Web of Science, Google Scholar, ProQuest, and Embase. The following Medical Subjects and Headings (Perona et al., 2004) terms and keywords were used: (1) “olive oil” or “virgin olive oil” or “refined olive oil” or “phenol” or “phenolic compound”; (2) “Malondialdehyde” or “MDA” or “Oxidized low‐density lipoprotein” or “OX‐LDL” or “Total Antioxidant Capacity” or “TAC” or “isoprostanes” or “ISOPS” or “Thiobarbituric acid reactive substances” or “TBARS” or “Protein Carbonyl” or “F2‐iso‐prostanes” or “8‐iso‐PGF2a” or “Uninduced conjugated diones” or “lipid hydroperoxide” or “Ferric reducing ability of plasma” or “FRAP”; (3) 1 & 2. To find more relevant papers, a hand search was performed on the references of related papers. All the studies published at any time till July 2021 with no language restriction were included.

### 2.2 Study selection and eligibility criteria

This systematic review and meta‐analysis were conducted in accordance with 2009 PRISMA checklist (Higgins & Green, 2011). Two different authors (ML and SM) screened the articles based on the title, abstract, and full text. Eligibility criteria were based on the PICOS format, where “population” included adults, “intervention” was phenolic content of olive oil, “Comparator” was olive oil with low or zero phenolic content, “outcomes” included oxidative stress biomarkers, and “study design” was randomized controlled trial studies. Articles having one of the following features were excluded: (1) animal study, editorial/letter to editor, or review article; (2) not being published in peer‐reviewed journals such as abstracts from conference proceedings, dissertations, or master's thesis; (3) studies with non‐RCT design; (4) having insufficient data; (5) interventions with single dosage; (6) using olive oil in combination with other ingredients; (7) using refined olive oil instead of low‐phenol olive oil as the control group.

### 2.3 Data extraction

Three authors (ML, SM, and SMDR) were responsible for extracting the data and the following data were extracted from each related article: first author's name, year, sample size, age, sex, study design, study duration, intervention dosage, health status, and outcomes. For effect size calculation, means and standard deviations (SDs) or standard errors (SEs) of ox‐LDL, MDA, and FRAP were extracted.

In total four papers assessed the effect of two different dosages of phenolic olive oil (Moreno‐Luna et al., 2012; Moschandreas et al., 2002; Silva et al., 2015; Vissers et al., 2001). The high‐ and low‐dosage of phenols in these studies were as follows: 546 mg/kg vs. approximately zero in Luna et al. study, 286 vs. 18 mg/kg in Silva et al study, and 308 vs. 43 mg/kg in Moschandreas et al. and Vissers et al. studies. The other four studies assessed the effects of three different dosages (Al‐Rewashdeh, 2010; Covas et al., 2006; Marrugat et al., 2004; Weinbrenner et al., 2004). These studies had three arms and the phenolic content of olive oil was categorized as low (varies from 0 to 132 mg/kg), medium (varies from 68 to 368 mg/kg), and high (differs from 150 to 753 mg/kg). Thus, we extracted 13, 13, and 6 effect sizes for the final analysis of ox‐LDL, MDA, and FRAP, respectively. Also, it should be noted that since four out of five studies used a cross‐over design, according to (Higgins et al., 2019), we calculated the effect sizes for this data by taking all assessments from the intervention and comparator periods and analyzed them as parallel group trials.

### 2.4 Quality assessment

The bias assessment of included studies was performed by Cochrane criteria (Higgins et al., 2019). Two authors (ML and SMDR) assessed the quality of the included studies regarding random sequence generation, allocation concealment, blinding, blinding of outcome assessor, incomplete outcome data, selective reporting, and risk of other biases. According to the Cochrane Handbook recommendation, studies were categorized as low risk, high risk, and unclear in each domain. Then, the overall quality of the studies was considered good, if all criteria were met, or only one criterion was rated unclear; fair, when one criterion was not met or two criteria were unclear; and poor, when two criteria were not met or more than two criteria were unclear.

### 2.5 Statistical analysis

We unified the scale of all outcomes and used U/L, μmol/L, and mmol/L for values of ox‐LDL, MDA, and FRAP, respectively. We used changes in mean and SD to estimate the effect size. If they were not reported directly, mean differences were computed by subtracting the mean of before‐ and after‐values for intervention and control groups. Then, SDs of mean differences were calculated by the following equation: $\mathrm{SD}=\sqrt{{\mathrm{SD}}_{\text{before}}^{2}+{\mathrm{SD}}_{\text{after}}^{2}-2*r*{\mathrm{SD}}_{\text{before}}*{\mathrm{SD}}_{\text{after}}}$, where “r” refers to the correlation between the before and after scores, which was calculated from data of included studies by using the following formula:

Mean was calculated by $\times =\frac{a+2m+b}{4}$ where “m” was median and “a” and “b” were low and high end of the range, respectively. The variance was calculated by the following equation: $s^{2}=\frac{1}{12}\;\left\{\frac{{\left(a-2m+b\right)}^{2}}{4}+{\left(b-a\right)}^{2}\right\}$ (Follmann et al., 1992). SE was converted to SD for the effect size calculation.

For heterogeneity assessment, both *I*‐squared and chi‐squared tests were used. In chi‐squared test, alpha value of less than 0.1 declared significant heterogeneity, and in *I*‐squared test, values <25% were considered as low heterogeneity, 25% to 50% as moderate heterogeneity, and more than 50% as high heterogeneity. For calculating the pooled effect size, random‐effect model (I–V heterogeneity, no standard) was applied. Confidence intervals (CIs) 95% were calculated for the weighted mean difference (WMD), and 0.05 or less was considered as significant levels. The funnel plot explains the publication bias using standard error as the measure of study size and ratio measures of treatment effect. All the statistical analyses were done using Stata version 11.0 software (Stata Corporation).

To assess the linear relationship between the phenolic content of olive oil and levels of oxidative indicators, we conducted two‐stage dose–response meta‐analysis (DRMA) which consisted of obtaining the regression coefficient of each individual study in the first stage and calculating the total coefficient by converging the weighted averages of the regression coefficients of individual studies in the second stage (Shim & Lee, 2019). We also showed the linearity and non‐linearity dose–response relationship for the effects of the phenolic content of olive oil graphically.

### 2.6 Quality of evidence

The quality of evidence for each outcome was assessed by the Grading of Recommendations Assessment, Development and Evaluation (GRADE) approach which contains the following domains: risk of bias, publication bias, imprecision of the results, inconsistency, indirectness of evidence, effect size, and dose–response relationship (Guyatt, Oxman, Akl, et al., 2011; Guyatt, Oxman, Schünemann, et al., 2011; Schünemann et al., 2008). Since the included studies in this meta‐analysis were randomized trials without important limitations, the baseline quality was considered as high. Then, the baseline score was downgraded or upgraded according to the mentioned domains. The criteria which we used to downgrade the quality included risk of bias, inconsistency, indirectness, imprecision, and publication bias. For risk of bias, we assessed the extent to which the high‐risk studies contribute toward the estimate of the magnitude of effect through study sample size. Inconsistency is considered as a not serious limitation when I^2^ was <50%, serious when I^2^ was between 50 and 75, and very serious for I^2^ > 75%. Indirectness was verified if our research directly compared the interventions which we were interested in and delivered to the populations in which we were interested. For imprecision, we assessed whether the sample size for the analysis met the optimal information size (OIS) criterion or not. For calculating OIS, we considered 0.05 and 0.2 as the α and β error thresholds, and minimally important difference (MID) as the Δ. MID was considered as one‐half standard deviation change in outcome measures (calculated from baseline values of participants included in a given analysis). Due to the small number of studies, publication bias analysis was not conducted. Effect size and presence of dose–response relationship were assessed to upgrade the quality of evidence. Standardized mean difference (SMD) of 0.2 to 0.49 was considered as a small effect (0 point); 0.5–0.79 moderate effect (+1 point); and ≥0.80 large effect (+2 point). The quality of evidence was categorized as high, moderate, low, and very low.

## 3 RESULTS

### 3.1 Study selection

**FIGURE 1:**
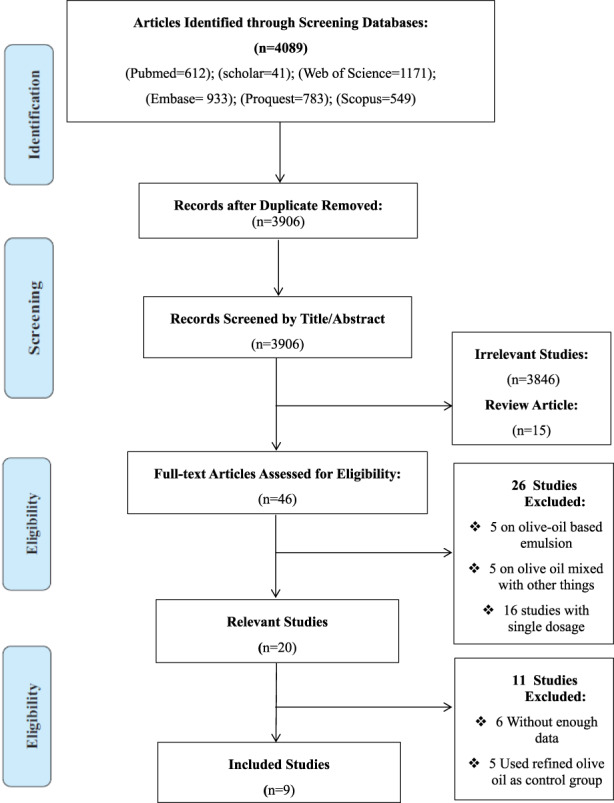
Flow Diagram of Database Searches and Study Selection.

Among 4089 articles, 46 full texts were assessed for inclusion and exclusion criteria (Figure 1). In total, thirty‐seven articles were excluded after the full‐text screening: 5 on olive oil‐based emulsion, 5 on olive oil in combination with other ingredients, 16 studies with single dosage, 6 studies with insufficient data, and 5 studies that used refined olive oil instead of low phenol olive oil as the control group. Finally, 9 studies were qualified to be enrolled in the meta‐analysis.

### 3.2 Characteristics of the included studies

The PICOS criteria of eligible studies are described in Table 1. Seven studies had cross‐over (CO) design, while only 1 study used parallel design. The study duration varied from 4 to 60 days. Mean age of the participants ranged from 18 to 75 years. Three studies were performed on males, 1 study on females and 4 studies on both genders. Among the eligible studies, 6 studies were done on healthy participants, 1 on healthy smokers, and 1 on individuals with hypertension.

**TABLE 1:** Characteristics and main outcome of the RCTs

Author (year)	Age (year)	Population	Sample size (LPC: MPC: HPC)	Design	Duration	Dosage (Mg/kg) (LPC: MPC: HPC)	Adverse effect	Result
Al‐Rewashdeh, 2010	37–50	healthy adults	25:25:25	CO	4 weeks	132:368:753	None	Plasma MDA level significantly decreased with increasing phenol content of olive oil.
Covas et al., 2006	20–60	healthy males	182:184:183	CO	3 weeks	2.7:164:366	None	Ox‐LDL level decreased linearly with increasing phenolic content.
Foshati et al., 2021	18–65	patients with depression	31:31	PA	<8 weeks (52 days)	….	None	Within and between group differences of MDA level were not significant.
Marrugat et al., 2004	57.4 ± 19.9	healthy males	30:30:30	CO	3 weeks	0:68:150	None	VOO with the HPC was more effective in protecting LDL from oxidation than LPC.
Moreno‐Luna et al., 2012	24–27	young Women with Mild Hypertension	24:24	CO	8 weeks	0:546	None	Only the polyphenol‐rich olive oil diet led to a significant decrease in ox‐LDL level.
Moschandreas et al., 2002	30 ± 9.13	normo‐lipidemic smokers	25:25	CO	3 weeks	43:308	None	Changes of MDA and FRAP was not significantly different between low‐ and high‐phenol olive oil diets.
Silva et al., 2015	18–75	healthy adults	34:29	PA	6 weeks	18:286	None	HPC OO does not lead to an improvement in cardiovascular health markers.
Vissers et al., 2001	18–58	healthy adults	46:46	CO	3 weeks	43:308	None	Mean of MDA and FRAP increased after the high‐phenol olive oil.
Weinbrenner et al., 2004	20–22	healthy males	12:12:12	CO	4 days	10:133:486	None	Short‐term consumption of OO decreased plasma ox‐LDL level.

Abbreviations: CO: Cross‐Over; HPC: High‐phenolic Content; LPC: Low‐phenolic Content; MPC: Medium‐phenolic Content; OO: Olive Oil; PA: Parallel; VOO: Virgin Olive Oil.

### 3.3 Quality assessment

Cochrane bias assessments of the included studies are summarized in Table S2. Six studies had good quality (Covas et al., 2006; Foshati et al., 2021; Marrugat et al., 2004; Moschandreas et al., 2002; Silva et al., 2015; Weinbrenner et al., 2004), and 3 others had fair quality (Al‐Rewashdeh, 2010; Moreno‐Luna et al., 2012; Vissers et al., 2001). Six studies had unclear risk of bias for allocation concealment (Al‐Rewashdeh, 2010; Marrugat et al., 2004; Moreno‐Luna et al., 2012; Moschandreas et al., 2002; Vissers et al., 2001; Weinbrenner et al., 2004), 3 studies for blinding of the outcome assessment (Al‐Rewashdeh, 2010; Covas et al., 2006; Vissers et al., 2001), and 1 for incomplete outcome data (Moreno‐Luna et al., 2012).

### 3.4 Meta‐analysis results

**FIGURE 2:**
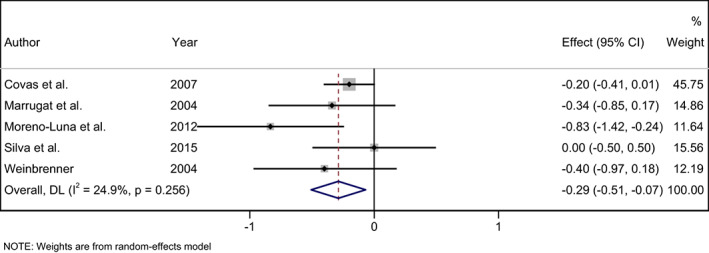
Forest plot of the effects of phenol rich olive oil on oxidized‐LDL level (OX‐LDL).

**FIGURE 3:**
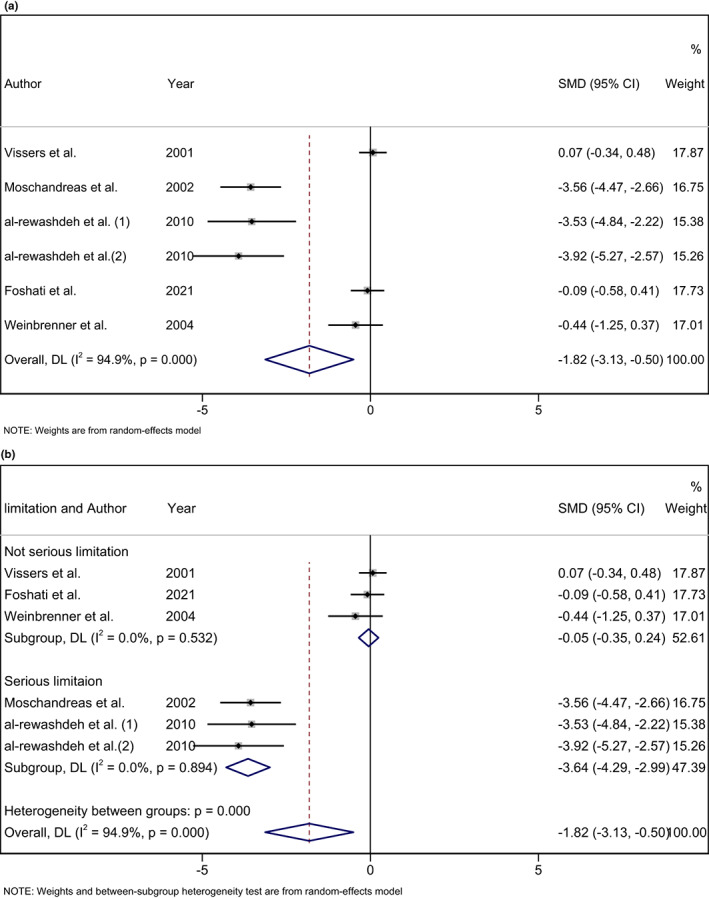
(a) Forest plot of the effects of phenol rich olive oil on Malondialdehyde (MDA) level. (b) Forest plot of the effects of phenol rich olive oil on Malondialdehyde (MDA) level based on subgroup analysis.

**FIGURE 4:**
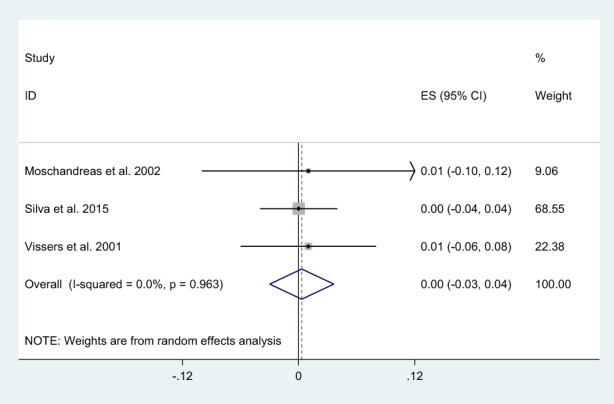
Forest plot of the effects of phenol rich olive oil on Ferric reducing ability of plasma (FRAP) level.

A significant decrease was observed in ox‐LDL level following consumption of phenol‐rich olive oil (WMD: −0.29 U/L; 95% CI: −0.51, −0.07) with non‐significant heterogeneity level (I^2^ = 24.9%, and *p* = .256) (Figure 2). Also, a significant decrease was found in MDA level following consumption of phenol‐rich olive oil (WMD: −1.82 μmoL/L; 95% CI: −3.13, −0.50) with high‐heterogeneity level (I^2^ = 94.9%, and *p* < .001) (Figure 3a). After subgroup analysis for MDA, the result was not significant for not serious limitation (SMD: −0.05, 95% CI: −0.35 to 0.24; I^2^: 0.0%, and *p* = .532), but significant for serious limitation (SMD: −3.64, 95% CI: −4.29 to −2.99; I^2^: 0.0%, and *p* = .894) (Figure 3b). The overall effect of phenol‐rich olive oil on FRAP level was not significant (WMD: 0.0 mmoL/L; 95% CI: −0.03, 0.04) with low‐heterogeneity level (I^2^ = 0.0%, and *p* = .963) (Figure 4).

### 3.5 Dose–response association between phenolic content of olive oil and ox‐LDL

**FIGURE 5:**
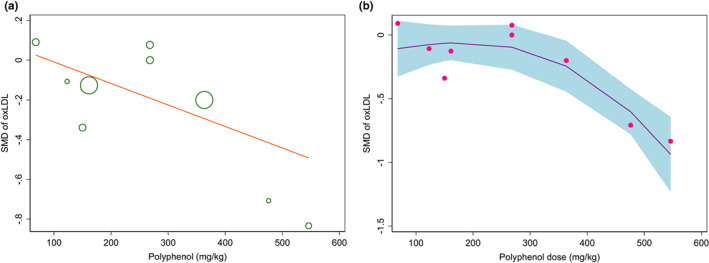
Dose–response analysis of the effect of phenolic content of olive oil on oxidized‐LDL level (ox‐LDL) (a). Linear model; (b). Non‐linear model.

Results of two‐stage random‐effect DRMA showed a linear relationship between the phenolic content of olive oil and Ox‐LDL based on the Wald test for linearity (*p* > .05) (Figure 5).

The phenolic content of olive oil significantly decreased SMD of ox‐LDL −0.019 per U/l (*p* = .024) (Figure 5).

### 3.6 Quality of meta‐evidence

The GRADE meta‐evidence rating indicated the low quality of evidence for ox‐LDL, and moderate quality for MDA and FRAP (Table S1).

## 4 DISCUSSION

This review summarized the high‐quality evidence provided by RCTs which assessed the effects of olive oil polyphenols on oxidative stress markers. The findings showed significant reductions in ox‐LDL and MDA levels following olive‐oil–rich phenols intake compared with the low‐phenol olive oil. However, no significant effect of phenol‐rich olive oil was observed on the FRAP level. The effect of olive oil phenols on ox‐LDL was dose‐dependent such that a higher concentration of phenol was related to more reduction in ox‐LDL.

Previous studies which assessed the effects of high‐phenol olive oil versus refined ones on oxidative stress biomarkers were inconsistent. In two studies, consumption of high phenols versus refined olive oil caused resistance to LDL oxidation in both patients with hyperlipidemia and peripheral vascular conditions (Masella et al., 2001; Ramirez‐Tortosa et al., 1999). Inversely, two other studies did not find any changes in markers of lipid peroxidation after the consumption of phenol‐rich olive oil in healthy adults (Holvoet et al., 1998; Weinbrenner et al., 2003). Since the participants in the last two studies were healthy and their biomarkers of oxidation were within the reference range, while the other two studies were done on unhealthy participants with uncontrolled markers of oxidation, the conflicting results can be attributed to the differences in the health status of the participants.

Also, two systematic reviews and meta‐analyses have assessed the effects of high‐polyphenol versus low‐polyphenol olive oil on cardiovascular disease risk factors (George et al., 2019; Hohmann et al., 2015). Accordingly, George et al., in 2018, found a significant moderate reduction in ox‐LDL as a pooled analysis of five studies (SMD: −0.44; 95% CI: −0.78, −0.10 μmol/L) (George et al., 2019), and Hohmann et al. (2015) reported a small significant reduction in ox‐LDL (*n* = 300; SMD −0.25; CI −0.50/0.00; *p* = .05) (Hohmann et al., 2015), using eight cross‐over trial including 355 participants. In addition, in some studies, the effect of olive oil on the reduction of MDA has been reported (Fang et al., 2008; Mansour et al., 2013).

Based on the subgroup analysis for the MDA, the results for not serious limitation were not significant but were significant for serious limitation. In a study by Vissers et al. indicated no significant effect of phenol‐rich extra virgin olive oil (EVOO) on MDA (Vissers et al., 2001). Moreover, a study by Moschandreas et al. found that EVOO did not significantly affect MDA (Moschandreas et al., 2002). Some of the studies mentioned earlier used refined olive oil as control groups and EVOO as the high‐polyphenol oil. When comparing phenol‐rich olive oil with refined olive oil, various aspects, such as variations in antioxidants and differences in polyphenol content, must be considered. Thus, their findings cannot be attributed to the phenolic content of olive oil by itself.

The plasma content of ox‐LDL is considered a risk factor for cardiovascular diseases (CVD) (Holvoet et al., 1998; Weinbrenner et al., 2003). The phenolic content of olive oil can modulate oxidative stress in human bodies and defend against chronic diseases (Holvoet et al., 1998; Weinbrenner et al., 2003). The findings of the present meta‐analysis can be justified by the phenolic content of olive oil, which keeps macronutrients (especially DNA and lipids) from carbonyl or oxidant reactions (Fitó et al., 2000). Also, they reduced lipid peroxidation via chelating superoxide radicals (Visioli et al., 1995). Moreover, in counteracting LDL oxidation, olive oil especially virgin olive oil has greater antioxidant capacity than refined one (Fitó et al., 2000; Visioli et al., 1995). Evidence proves that virgin olive oil intake increases the tocopherol and phenol content of LDL particles (Fitó et al., 2000; Visioli et al., 1995). In the present meta‐analysis, the lack of significant effect of olive oil phenols on FRAP level might be due to the non‐addressing postprandial effect. Visioli et al. demonstrated that, plasma clearance of olive oil phenols is fast, so phenol concentrations may fall to undetectable levels after 12 h (Visioli et al., 2000). Also, processing methods can influence polyphenol bioavailability and/or absorption. Accordingly, prolonged heat may completely deplete the polyphenol content of food stuffs (Brenes et al., 2002).

The present meta‐analysis has some strengths, including the comprehensive literature search and various analyses such as dose–response meta‐analysis, non‐linear dose–response analysis, also using low‐phenol olive oil instead of refined one as the control group. Besides, since we included prospective studies, risk of recall bias was removed. Furthermore, the present study used the latest method for conducting dose–response analysis as a Two‐stage random‐effect DRMA.

On the other hand, the present review has some limitations. The included studies showed substantial heterogeneity in the population, sample size, and follow‐up period. Besides, none of the included studies mention the ways of olive oil processing/ preparation. Moreover, only a few prospective studies reported the effects of different doses of olive oil on oxidative stress biomarkers, so the results should be interpreted with caution.

## 5 CONCLUSIONS

The finding of the present review supports the use of olive oil with high‐phenolic content to lower oxidative stress biomarkers in both patients and healthy participants. We observed some benefits of phenol‐rich olive oils on ox‐LDL and MDA levels. However, conducting further high‐quality trials with longer periods is suggested.

## FUNDING INFORMATION

“This research received no specific grant from any funding agency, commercial or not‐for‐profit sectors.”

## CONFLICT OF INTEREST

None.

## Supporting information


Table S1

Table S2

## Data Availability

The data that support the findings of this study are available on request from the corresponding author.
