# Supplementary material for: Effect of olive oil phenols on oxidative stress biomarkers: A systematic review and dose–response meta‐analysis of randomized clinical trials
Source: Food Sci Nutr. 2023 Mar 13;11(5):2393–402. doi: 10.1002/fsn3.3251 (PMC10171518; doi:10.1002/fsn3.3251)
Supplement: Supplementary file 1 — Table S1 [file FSN3-11-2393-s001.docx]

**Supplementary** **Table X** GRADE^1^ assessment of confidence in estimates of effect in randomized trials

| **Study** | **Risk of bias^2^** | **Inconsistency^3^** | **Indirectness** | **Imprecision^4^** | **Publication bias^5^** | **Effect size^6^** | **Dose-response** | **Certainty^7^** |
| --- | --- | --- | --- | --- | --- | --- | --- | --- |
| Ox-LDL | Not serious | Very serious | Not serious | Not serious | Serious | small | Yes | Low |
| MDA | Not serious | Very serious | Not serious | Serious | Not serious | Large | - | Moderate |
| FRAP | Not serious | Not serious | Not serious | Serious | Not serious | Trivial | - | Moderate |

^1^Grading of Recommendations Assessment, Development and Evaluation

^2^Risk of bias based on Cochrane Collaboration’s tool. Allocation concealment and selective reporting were the main limitations of most studies.

^3^ When *I*^2^ was <50% inconsistency considered as not serious limitation, >50 considered as serious and more than 75% considered as very serious limitation. In case the heterogeneity was >50 but the source of heterogeneity was found, it was considered as not serious.

^4^Serious limitations when the pooled sample size was less than optimal information size

^5^Based on the funnel plots, Egger or Begg’s; for the outcomes with small number of studies (n <10) risk of publication bias not formally assessed.

^6^Standardized mean difference of 0.2 to 0.49 was considered as small effect (0 point); 0.5-0.79 moderate effect (+1 point); and ≥0.80 large effect (+2 point).

^7^High quality: We are very confident that the true effect lies close to that of the estimate of the effect

Moderate quality: We are moderately confident in the effect estimate: The true effect is likely to be close to the estimate of the effect, but there is a possibility that it is substantially different
