# Supplementary material for: Effect of olive oil phenols on oxidative stress biomarkers: A systematic review and dose–response meta‐analysis of randomized clinical trials
Source: Food Sci Nutr. 2023 Mar 13;11(5):2393–402. doi: 10.1002/fsn3.3251 (PMC10171518; doi:10.1002/fsn3.3251)
Supplement: Supplementary file 2 — Table S2 [file FSN3-11-2393-s002.docx]

| **First author (year)** | **Random Sequence generation** | **Allocation concealment** | **Blinding** | **blinding of outcome assessment** | **Incomplete outcome data** | **Selective reporting** | **Other Biases** | **Overall Quality** |
| --- | --- | --- | --- | --- | --- | --- | --- | --- |
| Al-Rewashdeh et al. 2010. | low | unclear | low | unclear | low | low | low | Fair |
| Covas et al, 2006. | low | low | low | unclear | low | low | low | Good |
| Foshati et al, 2021. | low | low | low | unclear | low | low | low | Good |
| Marrugat et al, 2004. | low | unclear | low | low | low | low | low | Good |
| Moreno-Luna et al, 2012. | low | unclear | low | low | unclear | low | low | Fair |
| Moschandreas et al, 2002. | low | unclear | low | low | low | low | low | Good |
| Silva et al, 2015. | low | low | low | low | low | low | low | Good |
| Vissers et al, 2001. | low | unclear | low | unclear | low | low | low | Fair |
| Weinbrenner et al, 2004. | low | unclear | low | low | low | low | low | Good |

Table S1. Quality of included studies
